# Advanced maternal age and severe maternal morbidity in South Korea: a population-based cohort study

**DOI:** 10.1038/s41598-022-25973-x

**Published:** 2022-12-09

**Authors:** Juyeong Kim, Jin Young Nam, Eun-Cheol Park

**Affiliations:** 1 Department of Public Health, Sahmyook University, Seoul, Republic of Korea; 2 Department of Healthcare Management, Eulji University, Seongnam, Gyeonggi-do Republic of Korea; 3 Department of Preventive Medicine, Yonsei University College of Medicine, Seoul, Republic of Korea

**Keywords:** Preventive medicine, Risk factors, Epidemiology, Pregnancy outcome

## Abstract

To investigate the association between maternal age and severe maternal morbidity (SMM) in a Korean population. Data for cases of delivery between 2003 and 2019 were extracted from the Korean National Health Insurance Service-National Delivery Cohort. The main outcome was SMM, which was determined using the Center for Disease Control and Prevention’s algorithm. A generalized estimating equation model with a log link was performed for the relationship between SMM and maternal age adjusted for covariates. SMM occurred in 40,959/2,113,615 (1.9%) of delivery cases. Teenagers and women 35 years and older had an increased risk of SMM in both nulliparous and multiparous cases (ages 15–19: risk ratio (RR) 1.32, 95% confidence interval (CI) 1.15–1.46; ages 35–39: RR 1.24, 95% CI 1.21–1.28; ages 40–44: RR 1.57, 95% CI 1.50–1.64; and ages 45 or older: RR 2.07, 95% CI 1.75–2.44). Women aged 40 years and older had the highest rates of SMM. In singleton births as well as in nulliparous and multiparous cases, teenagers and women aged 35 years and older had a particularly high risk of SMM. Identifying and managing risk factors for SMM in these vulnerable age groups may improve maternal health outcomes.

## Introduction

Despite the overall decline in birth rate over the last decade, the number of births by women age 30 years and older have increased^1^. From 2009 to 2019, birth rates rapidly decreased by 13.4% for women aged 25–29 years, 5.7% for women aged 30–34 years, but increased for women aged 40 years and over in South Korea^2^. The socioeconomic environmental change of the development of assisted reproductive technology has enabled women to conceive at older ages. This trend of increasing births among older women is expected to last as reproductive technologies improve and become more accessible^3^.

Older women can experience adverse maternal health outcomes due to underlying comorbid conditions, such as diabetes, hypertension, and obesity^4^. Even accounting for their existing diseases, healthy women with advanced maternal age have reported increasing rates of pregnancy complication^5^. Therefore, both prevention and management of adverse health outcomes after pregnancy for the advanced maternal age group is needed.

To manage adverse health outcomes after pregnancy, severe maternal morbidity (SMM) is a useful indicator for both improvement and assessment of maternal health services^6^. SMM includes both psychological and physical conditions that result from or worsen during pregnancy and may adversely influence a woman’s health status. Women who develop SMM, compared to those who do not, experience increased medical costs, extended hospital stays, and potentially long-term adverse health outcomes^7^. Moreover, the identification of risk factors for SMM facilitate discovery of potentially hazardous health practices and may lower mortality^8^. Considering the increasing birth rate in older women despite a higher possibility of pregnancy complications, understanding the association between maternal age and SMM may help identify the risk factors for maternal deaths and provide focused care for vulnerable populations after delivery.

Previously, many studies exploring the association between maternal age and SMM have been conducted; however, the findings have been mixed. Some studies found a J-shaped association between maternal age and SMM, showing that the SMM of women aged 45 years or older had the highest risk followed by women aged 40–44 years and women in teenage years, compared to those in their mid to late 20 s^5,9–11^. On the other hand, some studies found only a higher risk of SMM among women aged 35 years or older^12–14^, 30 years or older^8,15^, or only among women aged 30–39 years^16^. One study found higher SMM risk in women aged 35 years or older and lower SMM risk among women aged 18 years or younger^17^. Thus, despite previous studies, the association between maternal age and SMM remains unclear. In addition, many studies have been conducted on Western populations; however, little information is available in the Korean context.

Therefore, we performed a nationwide population-based cohort study of childbirth cases in South Korea. The purpose of this study was to investigate the association between maternal age and SMM. In addition, this study aimed to determine the association between age and the risk of SMM by parity and the status of multiple births.

## Methods

### Data source

This population-based cohort study was collected from the National Health Information Database (NHID) between 2013 and 2019, which is composed of the health care utilization database, national health screening database, sociodemographic factors, and mortality for the entire South Korean population^18^. This data was formed by the National Health Insurance Service (NHIS), which is a single insurer covering the entire population of Korea^18^. The data linkage of the National Health Insurance could be made by using its unique resident registration numbers given to residents by the Korean government. The NHID uses de-identified join keys to substitute for personal identifiers to link these databases and to secure ethical clearance^18^. The healthcare utilization database, which is the biggest component of the NHID, is collected during the claim process of healthcare services and includes information on records of inpatient and outpatient medical usage (diagnosis according to the International Classification of Diseases, 10th Revision (ICD-10), length of stay, treatment costs, and services received and prescription records (drug code, days prescribed, daily dosage, etc.)^18^. The healthcare provider database contains the types of healthcare institutions, healthcare human resources, and equipment, and covers all health care institutions in Korea^18^.

This study used the NHIS delivery cohort, which was extracted using the NHID claims database. The NHIS delivery cohort included all childbirth cases in South Korean healthcare institutions. This study defined childbirth using diagnosis and procedure codes for pregnancies with maternal age between ages 13 − 50 years who had a delivery hospitalization. The study population was included for at least 280 days before childbirth through 6 weeks following childbirth between January 1, 2013, and November 19, 2019. We defined childbirth as any record of delivery hospitalization that included delivery-related diagnostic codes (ICD-10: O80, O81, O82, O83, and O84) and procedure codes for vaginal or cesarean delivery among pregnancies with more than 37 weeks of gestation. We excluded preterm births because the database did not have specific information regarding it, such as gestational age. Different preterm births statuses (e.g. very early preterm vs early preterm) may be associated with different risk of SMM. The total number of childbirths in 2013 to 2019 was 2,203,006. We excluded women who underwent delivery after November 19, 2019 (n = 19,960), those who had preterm births (n = 67,881), those with more than 42 days of length of hospital stay (n = 98), and those with no information on the childbirth healthcare institution (n = 1452). Therefore, a total of 2,113,615 childbirths between 2013 and 2018 were included in this study.

### Ethics statement

This research was approved by an ethical review from the Institutional Review Board of Eulji University in Korea (No. EU21-005), and all methods were performed in accordance with relevant guidelines and regulations. The requirement for informed consent was waived by the Institutional Review Board of Eulji University in Korea because all analysis used de-identified NHIS data.

### Severe maternal morbidity

To determine SMM, we utilized the SMM algorithms developed by the Centers for Disease Control and Prevention (CDC) and included women who had at least one of the 21 previously established ICD-10 diagnosis and procedure codes during childbirth hospitalization^19^. The original SMM algorithm was 25 SMM indicators based on the 9th revision of ICD^7^; however, in October 2015, the United States transitioned from the ICD-9 to ICD-10 diagnoses and procedures codes. The updated SMM algorithm represents serious complications of pregnancy or childbirth, such as eclampsia or acute renal failure, or procedures for the care of serious conditions, such as blood transfusion or hysterectomy. The 21 SMM indicators were composed of 16 diagnosis codes and five procedure codes.

### Maternal age

Maternal age at childbirth was the main exposure variable of interest. Maternal age was divided into 5-year age groups and used as a categorical variable in this study.

### Covariates

Possible confounders were selected based on previous studies as follows: household income level (quintile), type of insurance (self-employed insured, employee insured, and medical aid), and residential area (city and rural) were included as socioeconomic factors. Clinical factors included mode of delivery (spontaneous vaginal delivery, instrumental delivery, and cesarean section delivery), prenatal care (adequate, intermediate, or inadequate) using Kessner’s Adequacy of Prenatal Care Index^20^, parity (nulliparous or multiparous), twin birth status (singleton and twin birth), and maternal comorbidities (0, or 1 and more) using Howell’s study^21^. Other covariates included the type of hospitals, which were divided by the number of beds (beds < 30, 30 ≤ beds < 100, 100 ≤ beds < 500, and beds ≥ 500), hospital regions (Seoul, other metropolitan areas, small cities, and rural areas), and the calendar year of childbirth.

### Statistical analysis

We calculated the distribution of the general characteristics of the study population by maternal age at childbirth between 2013 and 2018. The association between maternal age and SMM during childbirth hospitalization was determined using a generalized estimating equation with a log link and robust standard errors, which estimated the adjusted risk ratio (RR) and 95% confidence intervals (CI). Moreover, this model was conducted for a stratified analysis by parity and twin birth status and tested for interactions. All statistical analyses were conducted using SAS (version 9.4; SAS Institute, Inc., Cary, NC, USA). The level of significance was set at p < 0.05.

## Results

Table 1 shows the general characteristics of the study population according to maternal age groups. Of the 2,113,615 women who had childbirth during the study period; 40,959 (1.9%) episodes of SMM occurred during the childbirth hospitalization period. In terms of maternal age, women aged 45 years or older had the highest incidence of SMM (5.3%), and the 2nd and 3rd highest incidences of SMM were in the teenage pregnancy group (3.4%), and the 40 to 44 years old pregnancy group (3.3%).

**Table 1:** General characteristics of study population according to maternal age.

	Maternal age, No. (%)															
	15–19 (n = 6409)		20–24 (n = 86,215)		25–29 (n = 406,587)		30–34 (n = 1,016,141)		35–39 (n = 520,305)		40–44 (n = 75,480)		45+ (n = 2478)		Total (n = 2,113,615)	
**Severe maternal morbidity**																
No	6191	(96.6)	84,473	(98.0)	399,728	(98.3)	998,209	(98.2)	508,680	(97.8)	73,029	(96.8)	2346	(94.7)	2,072,656	(98.1)
Yes	218	(3.4)	1742	(2.0)	6859	(1.7)	17,932	(1.8)	11,625	(2.2)	2451	(3.3)	132	(5.3)	40,959	(1.9)
**Household income level**																
Q1	2726	(42.5)	26,069	(30.2)	92,634	(22.8)	181,164	(17.8)	95,393	(18.3)	15,075	(20.0)	576	(23.2)	413,637	(19.6)
Q2	1564	(24.4)	28,108	(32.6)	124,201	(30.6)	228,924	(22.5)	96,389	(18.5)	14,835	(19.7)	539	(21.8)	494,560	(23.4)
Q3	1349	(21.1)	21,304	(24.7)	132,104	(32.5)	396,097	(39.0)	187,117	(36.0)	22,317	(29.6)	642	(25.9)	760,930	(36.0)
Q4	770	(12.0)	10,734	(12.5)	57,648	(14.2)	209,956	(20.7)	141,406	(27.2)	23,253	(30.8)	721	(29.1)	444,488	(21.0)
**Type of insurance**																
Self-employed insured	2573	(40.2)	31,137	(36.1)	90,813	(22.3)	177,792	(17.5)	111,552	(21.4)	23,365	(31.0)	973	(39.3)	438,205	(20.7)
Employee insured	2788	(43.5)	52,563	(61.0)	313,457	(77.1)	835,506	(82.2)	406,238	(78.1)	51,065	(67.7)	1,430	(57.7)	1,663,047	(78.7)
Medical aid	1048	(16.4)	2515	(2.9)	2317	(0.6)	2843	(0.3)	2515	(0.5)	1050	(1.4)	75	(3.0)	12,363	(0.6)
**Residential area**																
Seoul	686	(10.7)	9235	(10.7)	61,914	(15.2)	207,109	(20.4)	111,940	(21.5)	16,116	(21.4)	467	(18.9)	407,467	(19.3)
Other metropolitan	1587	(24.8)	21,330	(24.7)	102,731	(25.3)	262,599	(25.8)	135,475	(26.0)	19,243	(25.5)	613	(24.7)	543,578	(25.7)
Small city	3441	(53.7)	46,658	(54.1)	213,719	(52.6)	496,011	(48.8)	248,582	(47.8)	36,114	(47.9)	1229	(49.6)	1,045,754	(49.5)
Rural	695	(10.8)	8992	(10.4)	28,223	(6.9)	50,422	(5.0)	24,308	(4.7)	4007	(5.3)	169	(6.8)	116,816	(5.5)
**Mode of delivery**																
Spontaneous vaginal delivery	2345	(36.6)	28,270	(32.8)	123,260	(30.3)	309,422	(30.5)	142,353	(27.4)	15,628	(20.7)	377	(15.2)	621,655	(29.4)
Instrumental delivery	2483	(38.7)	32,110	(37.2)	145,154	(35.7)	322,568	(31.7)	140,567	(27.0)	16,103	(21.3)	401	(16.2)	659,386	(31.2)
Cesarean section delivery	1581	(24.7)	25,835	(30.0)	138,173	(34.0)	384,151	(37.8)	237,385	(45.6)	43,749	(58.0)	1700	(68.6)	832,574	(39.4)
**Adequacy of prenatal care**																
Adequate	4400	(68.7)	78,437	(91.0)	393,190	(96.7)	993,314	(97.8)	505,307	(97.1)	71,885	(95.2)	2,240	(90.4)	2,048,773	(96.9)
Intermediate	1803	(28.1)	6906	(8.0)	11,836	(2.9)	21,030	(2.1)	13,850	(2.7)	3263	(4.3)	218	(8.8)	58,906	(2.8)
Inadequate	206	(3.2)	872	(1.0)	1561	(0.4)	1797	(0.2)	1148	(0.2)	332	(0.4)	20	(0.8)	5936	(0.3)
**Parity**																
Nulliparous	5896	(92.0)	64,663	(75.0)	282,708	(69.5)	543,149	(53.5)	185,741	(35.7)	24,442	(32.4)	734	(29.6)	1,107,333	(52.4)
Multiparous	513	(8.0)	21,552	(25.0)	123,879	(30.5)	472,992	(46.6)	334,564	(64.3)	51,038	(67.6)	1744	(70.4)	1,006,282	(47.6)
**Status of multiple birth**																
Singleton	6381	(99.6)	85,760	(99.5)	403,495	(99.2)	1,003,165	(98.7)	510,891	(98.2)	74,384	(98.6)	2,451	(98.9)	2,086,527	(98.7)
Multiple	28	(0.4)	455	(0.5)	3092	(0.8)	12,976	(1.3)	9414	(1.8)	1096	(1.5)	27	(1.1)	27,088	(1.3)
**Maternal comorbidity**																
0	3862	(60.3)	48,834	(56.6)	218,633	(53.8)	416,105	(41.0)	191,510	(36.8)	25,251	(33.5)	795	(32.1)	904,990	(42.8)
1+	2547	(39.7)	37,381	(43.4)	187,954	(46.2)	600,036	(59.1)	328,795	(63.2)	50,229	(66.6)	1683	(67.9)	1,208,625	(57.2)
**Type of hospital (no. of beds)**																
500+	294	(4.6)	2029	(2.4)	12,608	(3.1)	46,119	(4.5)	32,857	(6.3)	6985	(9.3)	377	(15.2)	101,269	(4.8)
100–499	617	(9.6)	4896	(5.7)	22,240	(5.5)	67,274	(6.6)	45,781	(8.8)	9424	(12.5)	403	(16.3)	150,635	(7.1)
30–99	2616	(40.8)	39,386	(45.7)	200,893	(49.4)	512,092	(50.4)	254,096	(48.8)	33,698	(44.6)	985	(39.8)	1,043,766	(49.4)
< 30	2882	(45.0)	39,904	(46.3)	170,846	(42.0)	390,656	(38.5)	187,571	(36.1)	25,373	(33.6)	713	(28.8)	817,945	(38.7)
**Hospital region**																
Seoul	724	(11.3)	9559	(11.1)	63,153	(15.5)	211,657	(20.8)	119,366	(22.9)	18,096	(24.0)	559	(22.6)	423,114	(20.0)
Other metropolitan	1984	(31.0)	24,656	(28.6)	117,562	(28.9)	294,779	(29.0)	149,374	(28.7)	21,221	(28.1)	707	(28.5)	610,283	(28.9)
Small city	3635	(56.7)	50,966	(59.1)	223,964	(55.1)	506,620	(49.9)	249,954	(48.0)	35,824	(47.5)	1,190	(48.0)	1,072,153	(50.7)
Rural	66	(1.0)	1034	(1.2)	1908	(0.5)	3085	(0.3)	1611	(0.3)	339	(0.5)	22	(0.9)	8065	(0.4)
**Year**																
2013	1494	(23.3)	17,533	(20.3)	81,283	(20.0)	204,243	(20.1)	78,816	(15.2)	12,091	(16.0)	370	(14.9)	395,830	(18.7)
2014	1392	(21.7)	16,523	(19.2)	76,362	(18.8)	196,015	(19.3)	83,172	(16.0)	12,171	(16.1)	363	(14.7)	385,998	(18.3)
2015	1100	(17.2)	16,156	(18.7)	74,285	(18.3)	190,326	(18.7)	92,315	(17.7)	12,763	(16.9)	388	(15.7)	87,333	(18.3)
2016	1039	(16.2)	14,589	(16.9)	67,990	(16.7)	168,352	(16.6)	94,125	(18.1)	12,953	(17.2)	457	(18.4)	359,505	(17.0)
2017	796	(12.4)	12,076	(14.0)	59,039	(14.5)	140,316	(13.8)	89,950	(17.3)	13,217	(17.5)	467	(18.9)	315,861	(14.9)
2018	588	(9.2)	9338	(10.8)	47,628	(11.7)	116,889	(11.5)	81,927	(15.8)	12,285	(16.3)	433	(17.5)	269,088	(12.7)

Table 2 presents the association between maternal age and SMM, adjusted for all covariates. The risk of SMM by maternal age presented a J-shape based on the 25 to 29 age group. The maternal group aged 45 years and older had an approximately twofold higher risk of SMM than those aged 25–29 years (RR 2.17, 95% CI 1.06–2.44). Women who were 40–44 years old had an approximately 1.6-fold higher risk of SMM (RR 1.57, 95% CI 1.50–1.64), and those who were teenaged had an approximately 1.3-fold higher risk of SMM (RR 1.32, 95% CI 1.15–1.51) compared to the reference group. The risk of SMM was higher in women who had instrumental delivery and cesarean section delivery, in those who had inadequate prenatal care, those who were nulliparous, those who had twin births, those who had maternal comorbidities, and those who had childbirth in the tertiary hospital, compared to the reference group.

**Table 2:** Relative risk for the relationship between maternal age and SMM adjusted for all covariates.

	SMM	
	RR	(95% CI)
**Maternal age (years)**		
15–19	1.32	(1.15–1.51)
20–24	1.12	(1.07–1.18)
25–29	1.00	
30–34	1.03	(1.00–1.06)
35–39	1.24	(1.21–1.28)
40–44	1.57	(1.50–1.64)
45 +	2.07	(1.75–2.44)
**Household income level**		
Q1	1.09	(1.06–1.12)
Q2	1.10	(1.07–1.13)
Q3	1.06	(1.04–1.09)
Q4	1.00	
**Type of insurance**		
Self-employed insured	1.11	(1.08–1.13)
Employee insured	1.00	
Medical aid	2.09	(1.92–2.28)
**Residential area**		
Seoul	1.00	
Other metropolitan	1.09	(1.04–1.15)
Small city	1.18	(1.14–1.22)
Rural	1.20	(1.14–1.27)
**Mode of delivery**		
Spontaneous vaginal delivery	1.00	
Instrumental delivery	1.61	(1.57–1.66)
Cesarean section delivery	1.56	(1.52–1.60)
**Adequacy of prenatal care**		
Adequate	1.00	
Intermediate	1.45	(1.38–1.52)
Inadequate	1.54	(1.33–1.78)
**Parity**		
Nulliparous	1.34	(1.31–1.36)
Multiparous	1.00	
**Status of multiple birth**		
Singleton	1.00	
Multiple	2.04	(1.95–2.13)
**Maternal comorbidity**		
0	1.00	
1+	1.31	(1.29–1.34)
**Type of hospital (no. of beds)**		
500+	4.33	(4.20–4.47)
100–499	2.44	(2.36–2.51)
30–99	1.00	
< 30	1.04	(1.02–1.07)
**Hospital region**		
Seoul	1.00	
Other metropolitan	1.23	(1.18–1.29)
Small city	1.10	(1.06–1.14)
Rural	1.18	(1.01–1.38)
**Year**		
2013	1.00	
2014	0.96	(0.93–0.99)
2015	1.02	(0.99–1.05)
2016	1.00	(0.97–1.04)
2017	0.95	(0.92–0.98)
2018	0.97	(0.94–1.00)

Adjusted for household income level, type of insurance, residential area, mode of delivery, adequacy of prenatal care, parity, status of multiple birth, maternal comorbidity, type of hospital, hospital region, and year.

We also performed a stratified analysis of parity and twin birth status, which confirmed an interaction between maternal age and each stratified variable on SMM. In parity, women aged 40 years and older had approximately 1.5-folds higher risk of SMM in both nulliparous and multiparous women compared to the reference group (nulliparous: 40–44 years: RR 1.47, 95% CI 1.38–1.59, 45 + : RR 1.48, 95% CI 1.04–2.06; multiparous: 40–44 years: RR 1.57, 95% CI 1.47–1.69; 45 + : RR 2.18, 95% CI 1.79–2.64). In twin birth status, women aged 40 years and older had a 1.59- and 2.1-fold higher risk of SMM in singleton, respectively (40–44 years: RR 1.59, 95% CI 1.52–1.67; 45 + : RR 2.06, 95% CI 1.74–2.44); however, the only statistically significant association was between women aged 45 and older and the risk of SMM with multiple births (RR 2.39, 95% CI 1.11–5.13) (Table 3).

**Table 3:** The relationship between maternal age and severe maternal morbidity stratified by parity and status of multiple births.

	Severe maternal morbidity												
	15–19		20–24		25–29	30–34		35–39		40–44		45+	
	RR	(95% CI)	RR	(95% CI)	RR	RR	(95% CI)	RR	(95% CI)	RR	(95% CI)	RR	(95% CI)
**Parity**													
Nulliparous	1.44	(1.24–1.66)	1.12	(1.06–1.19)	1.00	1.08	(1.04–1.11)	1.25	(1.21–1.30)	1.48	(1.38–1.59)	1.47	(1.04–2.06)
Multiparous	1.76	(1.14–2.73)	1.22	(1.09–1.36)	1.00	0.97	(0.91–1.02)	1.24	(1.17–1.30)	1.57	(1.47–1.69)	2.18	(1.79–2.64)
**Status of multiple birth**													
Singleton	1.32	(1.15–1.52)	1.12	(1.06–1.18)	1.00	1.03	(1.00–1.06)	1.26	(1.22–1.30)	1.59	(1.52–1.67)	2.06	(1.74–2.44)
Multiple	1.15	(0.47–2.81)	1.26	(0.90–1.75)	1.00	0.98	(0.84–1.13)	1.04	(0.89–1.21)	1.13	(0.89–1.43)	2.39	(1.11–5.13)

Adjusted for household income level, type of insurance, residential area, mode of delivery, adequacy of prenatal care, parity, status of multiple birth, maternal comorbidity, type of hospital, hospital region, and year, except for the stratum itself.

## Discussion

Using nationwide cohort data, this study examined the association between maternal age and SMM. We found that the risk curve of SMM by maternal age exhibits a J-shape, whereby younger and older women have the highest rates of SMM, with the maternal group of ages 45 years and older having the highest rate. Furthermore, women aged 45 years and older had a particularly high risk for SMM among nulliparous and multiparous women as well as in singleton and twin births.

Many studies have assessed the association between maternal age and SMM, but not in Asian populations. The findings of this study are consistent with those of a previous study addressing a J-shaped relationship between maternal age and SMM^5,9–11^. Considering the limited information about Asian studies regarding this issue, this study added evidence to the association between maternal age and SMM based on data from a nationally representative Korean population.

The study findings can be explained as follows. First, the risk curve of SMM by maternal age exhibits a J-shape whereby younger and older women have higher rates of SMM, with maternal group aged 45 years and older having the highest rate. Baseline factors associated with aging, including hypertension, diabetes, or lower cardiac output, could make older mothers more vulnerable to physiological changes during pregnancy, thereby increasing the risk of SMM^9^. In addition, a previous study found that women of ages 15–17 were more likely to have eclampsia than any other study group^5^. Considering that eclampsia is one of the illnesses comprising SMM^19^, a higher SMM among women aged 15–19 years than among women aged 25–29 years could be possible. Second, the stratified analysis of parity indicated that the risk of SMM was high in both nulliparous and multiparous women, and the risk was particularly high among multiparous women aged 45 years and older, compared to that in nulliparous and multiparous women. A previous study noted that pregnancy complications and adverse pregnancy outcomes were associated with increasing parity^15,22^, and this might be a factor that worsens SMM among older mothers. Finally, the stratified analysis of twin birth status indicated that the risk of SMM was high in both singleton and twin births, and the risk of SMM was particularly high among women aged 45 years and older. Previously, the risk of SMM during pregnancy and postpartum periods was found more frequently among women with twin births than among singleton pregnancies^23^. In addition, in those with a maternal age of 40 years and older, multiple births were found to be one of the strongest risk factors for SMM^15^. These factors may lead to the most adverse SMM.

South Korea has suffered from the problem of low fertility. The total fertility rate is 0.84 in 2020, which is the lowest fertility rate among the Organisation for Economic Co-operation and Development (OECD) countries^24^. Numerous professionals have pointed out that later marriage and advanced maternal age have contributed to this problem. The average ages of first marriage and first childbirth were 31.1 and 32.6 years in 2021, respectively, which were significantly higher compared to 26.5 and 27.7 years, respectively, reported in 2000^25,26^. The average age of childbirth was 33.4 years, which has increased by 4.14 years since 2000^26^. These social phenomena affect advanced maternal age, which might also affect adverse maternal health outcomes, such as increasing the maternal mortality ratio or the risk of SMM. Therefore, it is necessary to monitor whether maternal age affects health outcomes and to make additional efforts to improve maternal health status.

This study had several strengths. To the best of our knowledge, this is the first study to examine the association between maternal age and SMM based on a nationally representative Korean delivery cohort. Furthermore, this study encompasses a very large population and long-term study because this database includes 18 years of follow up data for all Korean women who have delivered. Second, this study provides meaningful results in that not only nulliparous but also multiparous women can affect their risk of SMM by being of advanced maternal age, especially for women aged 40 years and older. This might be of value to countries which have late childbirth trends and may inform them how to avoid preventable maternal deaths or adverse maternal health outcomes. Third, this study considered several risk factors, including socioeconomic status, obstetric status, and provision factors. However, this study does have some limitations. First, the incidence of SMM might have been underestimated. Medical practitioners could under-report the incidence of SMM in medical records to report better quality indicators of services. Second, the observational nature of our study leaves room for residual confounding and other potential sources of bias, such as maternal BMI, or alcohol or smoking habits. We could not control some risk factors that were not included in this database. In addition, parity might not have been adequately adjusted for because of the limited database. The grand multiparous group (e.g. experienced five and more childbirths) and advanced maternal age might have a correlation; however, the database only contained information on the status of nulliparous or multiparous. Therefore, we could not completely adjust for multiparity, especially grand multiparity.

This study has several clinical implications. As it has been confirmed that SMM is higher in teens and older women than in women in their late 20s, additional efforts to identify and manage risk factors for SMM in vulnerable age groups are needed. In addition, though it is classified as a risk group from the age of 35 years or older, the relatively young 35–39 year-old group was identified as a group with high SMM regardless of whether it was nulliparous or not. Considering these results, it would be necessary to highlight the importance of optimal childbirth age to prevent adverse maternal health outcomes in order to ensure a healthy life after childbirth.

## Conclusion

The risk of SMM by maternal age exhibits a J-shape whereby younger and older women have the highest rates of SMM, with maternal group ages 40 years and older having the highest. Furthermore, women aged 40 years and older had a particularly high risk for SMM in nulliparous and multiparous women as well as in singleton and twin births. To improve maternal health outcomes, policymakers need more specific efforts to identify and manage risk factors for SMM in these vulnerable age groups.

## Data Availability

The datasets generated during and/or analyzed during the current study are available from the corresponding author on a reasonable request.
